# Predictive Value of Preresidency Academic Metrics on Resident Publication Potential

**DOI:** 10.1002/oto2.34

**Published:** 2023-02-17

**Authors:** Linda X. Yin, Dominic J. Catalano, Susan E. Bisco, Christine M. Lohse, Matthew L. Carlson, Janalee K. Stokken

**Affiliations:** ^1^ Department of Otolaryngology–Head and Neck Surgery Mayo Clinic Rochester Minnesota USA; ^2^ Department of Quantitative Health Sciences Mayo Clinic Rochester Minnesota USA

**Keywords:** academic, otolaryngology, potential, publication, research, residency

## Abstract

**Objective:**

Otolaryngology residency is highly competitive, and applicant academic metrics are scrutinized. The predictive value of preresidency academic metrics on applicants’ future research productivity and career aspirations remains largely undefined.

**Study Design:**

Retrospective cohort study

**Setting:**

Academic otolaryngology department, 2014 to 2015.

**Methods:**

Applicant demographics, publication history, and United States Medical Licensing Examination (USMLE) scores were downloaded from Electronic Residency Application Service archives. Publications during residency were tallied from all PubMed articles indexed between July 1, 2015 and June 30, 2020. Postresidency career paths were examined by 2 investigators (D.J.C. and L.X.Y.) using Google searches with an emphasis on program websites, Doximity, and LinkedIn profiles. Associations with publication potential and postresidency positions were evaluated with Spearman rank correlation coefficients and Kruskal‐Wallis, Wilcoxon rank sum, and *χ* ^2^ tests.

**Results:**

Of 321 applicants, 226 (70%) matched, and 205 (64%) completed residency by June 2020. Matched residents published a median of 4 (range: 0‐41) manuscripts during residency. USMLE scores, Alpha Omega Alpha status, and the number of preresidency publications did not significantly correlate with publication potential during residency. The number of research experiences had a significant positive correlation with publications during residency (*p* < 0.001). Asian race (*p* = 0.002) and geographical region of residency (*p* < 0.001) also had significant associations with publication potential. Of the 205 graduates, 118 (58%) enrolled in fellowship. Age and female sex (74% vs 48%; *p* = 0.002) were the only factors significantly associated with pursuing a fellowship.

**Conclusion:**

In otolaryngology, not all preresidency academic metrics are associated with publication potential during residency or propensity for fellowship training. Programs should not use academic metrics alone to predict an applicant's future research productivity or career trajectory.

Otolaryngology is consistently one of the most competitive surgical subspecialties in the residency match. The popularity of the field, the limited availability of training positions, and the anxiety of going unmatched have created a bottleneck effect in the application process.^1^, ^2^ There has been a consistent increase in the number of prospective applications per program, which strains the ability of training programs to provide each application with a truly holistic review—a crisis that has only partially been mitigated with the initiation of a signaling system.^3^

To efficiently vet applicants, many programs have used the United States Medical Licensing Examination (USMLE) scores, Alpha Omega Alpha (AOA) status, medical school ranking, and prior research experience.^4^ Nearly half of US otolaryngology programs admit to using USMLE Step 1 cutoff scores when reviewing applications.^5^ Medical school tier is often associated with resident performance, although lower‐ranked schools have been shown to correlate with higher performance.^6^ AOA status and publications are significantly associated with odds of matching into otolaryngology residency.^7^ Yet the reliance on these traditional academic markers of success may be misguided, as studies have shown these may not accurately predict clinical success or acquisition of surgical skills during training.^8^, ^9^, ^10^

The impact of these traditional selection metrics on performance during residency and beyond has not been adequately studied through the lens of publication potential and the pursuit of fellowship after graduation. Research has become a required component of otolaryngology training^11^ and acts as both a measure of academic success after training.^12^ The expectation among otolaryngology programs for applicant research experience^4^ has fueled the significant rise in preresidency research activity and participation in a research gap year.^13^ It remains unclear whether this reflects the dedication to research and future career aspiration, or simply a short‐term goal based on the perception that research experience is a prerequisite to a successful match.

In this study, we aim to explore associations between academic metrics in residency applications and the publication potential and career aspirations of matched applicants in otolaryngology. We hypothesize that USMLE Step scores and AOA status do not predict an applicant's propensity to publish during residency, enroll in fellowship, or enter academic or private practice after graduation.

## Methods

### Participant Identification

All applicants to the authors’ Accreditation Council for Graduate Medical Education accredited 5‐year otolaryngology training program in the 2014 to 2015 application cycle were identified using the Electronic Residency Application Service (ERAS). This study was deemed exempt by the Mayo Clinic Institutional Review Board (IRB 20‐001640). Full applications including sections on applicant demographics, publication and presentation history, work experience, academics, and USMLE Step 1 and Step 2 scores were downloaded from the ERAS archives.

### ERAS Data Extraction

Demographics extracted from ERAS included birthdate, sex, race and ethnicity, birthplace, participation in the couples match, and citizenship status. Race and ethnicity were categorized as Asian, black, Hispanic, Middle Eastern, Mixed, Native American, white, or “prefer not to say” as reported in ERAS. Birthplace was categorized as within the United States or in a foreign country. Within the United States, birthplace was further categorized as regions in the Northeast, Southeast, Southwest, Midwest, or West. Medical schools were classified as US allopathic, US osteopathic, or foreign. Applicants from US medical schools were further subdivided by the regions summarized above. USMLE board scores were extracted. Applicant AOA status was categorized as AOA, non‐AOA, or AOA is not announced/awarded at the applicant's home school. Advanced degrees were noted. An applicant's undergraduate field of study was categorized as sciences and health, arts and humanities, business, multiple majors, or unknown. Preresidency research was measured as the number of posters, oral presentations, research experiences, total publications, first‐author publications, and submitted publications reported on ERAS.

### Publication Potential and Career Trajectory

All matched applicants were followed to determine the residency program they attended and their career placement immediately after graduation. Several internet sources were used to determine an applicant's ultimate training program and postresidency career trajectory, including Google searches with an emphasis on Doximity, LinkedIn, published fellowship match results, and academic department websites. These searches were performed by 2 independent reviewers (D.J.C. and L.X.Y.). Residency programs were categorized by US region as summarized above. Residency programs were ranked using the Doximity Residency Navigator function, sorting programs by “research output.” Of note, this methodology for finding career trajectory only confirms which applicants ultimately matched and enrolled into fellowship programs, but does not provide information on which applicants applied to but were not selected for fellowships, nor does it provide information on which applicants ultimately graduated from fellowship training. Publication potential during residency was defined as the number of PubMed‐indexed publications for each applicant between July 1, 2015 and June 30, 2020. PubMed searches were conducted by 2 independent reviewers (D.J.C. and L.X.Y.) by querying each applicant by Last Name, First Name as well as Last Name, FM where F is the first name initial and M is the middle name initial. Publications were then reviewed in detail to identify the authors’ institutional affiliations, confirming each PubMed‐indexed study as authored by the applicant rather than by another author with a similar name. An attempt was made to identify the *H*‐index of applicants using Google Scholar, but most applicants did not have profiles and therefore the *H*‐index could not be used as a metric to measure publication potential in this cohort.

### Statistical Analysis

Continuous features were summarized using medians, interquartile ranges, and ranges, and categorical features were summarized using frequencies and percentages. Features of matched and unmatched applicants were compared using Wilcoxon rank sum and *χ* ^2^ tests. Associations between the number of publications during residency and ERAS features were evaluated using Spearman rank correlation coefficients, Kruskal‐Wallis tests, or Wilcoxon rank sum tests. Associations between postresidency career path (private practice, academic general practice, or fellowship) and ERAS features were evaluated using Kruskal‐Wallis, *χ* ^2^, or Fisher exact tests. Statistical analyses were conducted using SAS version 9.4 (SAS Institute). All tests were 2‐sided and *p* < 0.05 were considered statistically significant.

## Results

### Applicant Demographics and Matched Applicants

In the 2014 to 2015 application cycle, the residency program received 337 applications; 16 were excluded due to incomplete information received from ERAS, leaving 321 applicants for study (Table 1). Most applicants self‐identified as male (64%) and white (58%). The majority were born in the United States (77%) and current US citizens (93%), and 226 (70%) matched into otolaryngology (Table 2).

**Table 1:** Summary of 2014 to 2015 Otolaryngology Residency Applicants, N = 321

Feature^a^	
Age at time of application (y)	26 (25‐28; 23‐41)
Sex	
Male	206 (64)
Female	115 (36)
Race and ethnicity (N = 307)	
Asian	84 (27)
Black	9 (3)
Hispanic	14 (5)
Middle Eastern	16 (5)
Mixed	3 (1)
Native American	2 (1)
White	179 (58)
Couples match (N = 294)	18 (6)
Birthplace	
United States	247 (77)
Outside of the United States	74 (23)
Region of US birthplace (N = 247)	
Northeast	48 (19)
Southeast	49 (20)
Southwest	23 (9)
Midwest	74 (30)
West	53 (21)
US citizen	300 (93)
Medical school	
US MD	297 (93)
US DO	2 (1)
Outside of the United States	22 (7)
Region of US medical school (N = 299)	
Northeast	61 (20)
Southeast	95 (32)
Southwest	26 (9)
Midwest	88 (29)
West	29 (10)
USMLE Step 1	247 (238‐255; 193‐272)
USMLE Step 2 (N = 272)	253 (244‐262; 192‐282)
AOA	
No	145 (45)
Yes	107 (33)
Not awarded at school	69 (22)
Advanced degree	58 (18)
Undergraduate field of study (N = 308)	
Sciences and health	228 (74)
Arts and humanities	28 (9)
Business	10 (3)
Multiple	42 (14)
Number of poster presentations	2 (1‐4; 0‐15)
Number of oral presentations	1 (0‐2; 0‐14)
Research experience	4 (3‐6; 0‐13)
Number of reported total publications	1 (0‐2; 0‐14)
Number of reported first‐author publications	0 (0‐0; 0‐9)
Number of reported unpublished submissions	1 (0‐2; 0‐11)

Abbreviations: AOA, Alpha Omega Alpha; IQR, interquartile range; USMLE, United States Medical Licensing Examination.

^a^ Features were summarized with median (IQR; range) or n (%).

**Table 2:** Comparisons of 2014 to 2015 Residency Applicants by Match Success, N = 321

Feature^a^	Unmatched (n = 95)	Matched (n = 226)	*p* value^b^
Age at time of application (y)	27 (25‐30; 23‐41)	26 (25‐28; 23‐40)	**0.036**
Sex			
Male	65 (68)	141 (62)	0.30
Female	30 (32)	85 (38)	
Race and ethnicity (N = 307)			
Asian	30 (34)	54 (25)	0.06
White	42 (48)	137 (63)	
All others	16 (18)	28 (13)	
Couples match (N = 294)	4 (4)	14 (7)	0.41
Birthplace			
United States	57 (60)	190 (84)	<**0.001**
Outside of the United States	38 (40)	36 (16)	
Region of US birthplace (N = 247)			
Northeast	11 (19)	37 (19)	0.49
Southeast	16 (28)	33 (17)	
Southwest	4 (7)	19 (10)	
Midwest	15 (26)	59 (31)	
West	11 (19)	42 (22)	
US citizen	80 (84)	220 (97)	**<0.001**
Medical school			
United States	73 (77)	226 (100)	<**0.001**
Outside of the United States	22 (23)	0	
Region of US medical school (N = 299)			
Northeast	17 (23)	44 (19)	0.63
Southeast	25 (34)	70 (31)	
Southwest	7 (10)	19 (8)	
Midwest	20 (27)	68 (30)	
West	4 (5)	25 (11)	
USMLE Step 1	239 (224‐248; 193‐268)	249 (241‐256; 212‐272)	**<0.001**
USMLE Step 2 (N = 272)	242 (233‐256; 192‐270)	256 (247‐263; 215‐282)	**<0.001**
AOA			
No	46 (48)	99 (44)	<**0.001**
Yes	14 (15)	93 (41)	
Not awarded at school	35 (37)	34 (15)	
Advanced degree	19 (20)	39 (17)	0.56
Undergraduate field of study (*N* = *308*)			
Sciences and health	68 (83)	160 (71)	**0.032**
All others	14 (17)	66 (29)	
Number of poster presentations	1 (0‐3; 0‐15)	2 (1‐4; 0‐11)	**0.002**
Number of oral presentations	1 (0‐2; 0‐10)	1 (0‐2; 0‐14)	0.13
Research experience	4 (1‐5; 0‐9)	5 (3‐6; 0‐13)	**<0.001**
Number of reported total publications	1 (0‐2; 0‐14)	1 (0‐2; 0‐9)	0.89
Number of reported first‐author publications	0 (0‐0; 0‐9)	0 (0‐1; 0‐5)	0.70
Number of reported unpublished submissions	0 (0‐1; 0‐11)	1 (0‐2; 0‐8)	**0.006**

Bolded values indicate statistical significance, defined as *p* < 0.05.

Abbreviations: AOA, Alpha Omega Alpha; IQR, interquartile range; USMLE, United States Medical Licensing Examination.

^a^ Features were summarized with median (IQR; range) or n (%).

^b^ *p* values were obtained from Wilcoxon rank sum tests for continuous features and *χ* ^2^ tests for categorical features.

### Academic Potential of Matched Applicants

**Figure 1:**
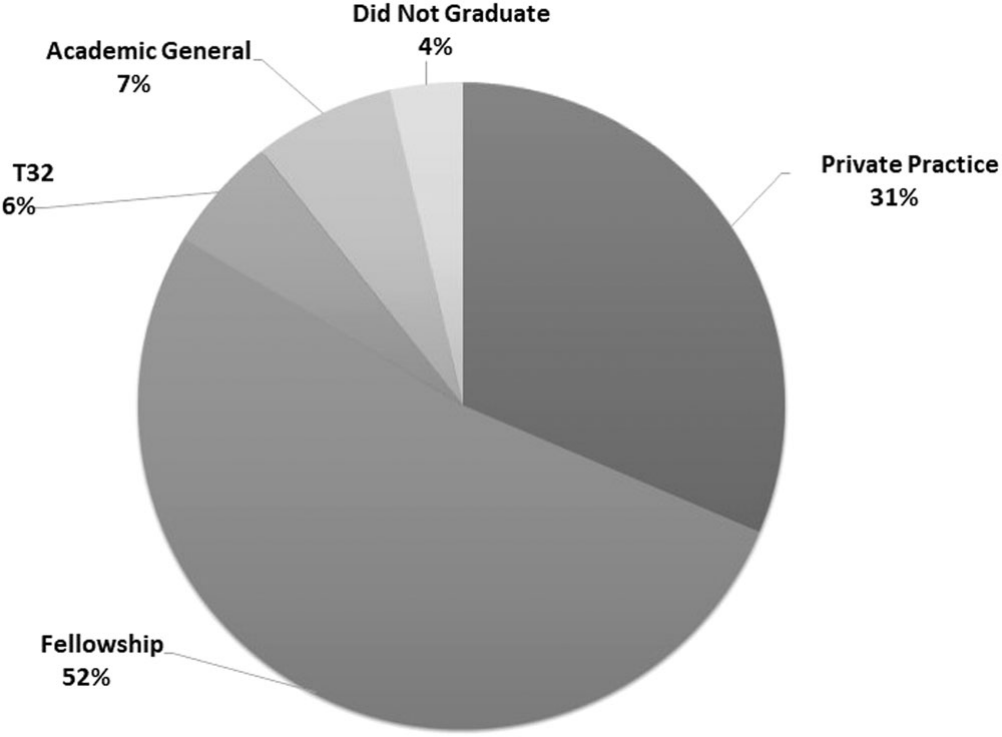
Distribution of career trajectory after 5 years of otolaryngology training in the 2014 to 2015 class of matched applicants.

During residency, matched applicants published a median of 4 papers (range: 0‐41; Table 3). After 5 years of residency training, just over half of matched otolaryngology residents enrolled in further subspecialty training in fellowship (n = 118, 52%). An additional 71 (31%) went into private practice, 16 (7%) entered general academic or academic‐affiliated practice, and 13 (6%) remained in training as T32 research track residents. Eight (4%) of matched residents did not graduate or complete residency by the end of the academic year in 2020 (Figure 1).

**Table 3:** Academic Potential of 2014 to 2015 Residency Applicants Who Matched, N = 226

Feature^a^	
Number of publications during residency	4 (2‐7; 0‐41)
Region of residency	
Northeast	48 (21)
Southeast	56 (25)
Southwest	18 (8)
Midwest	69 (31)
West	35 (15)
Residency Doximity research ranking	
1‐20	68 (30)
21‐50	76 (34)
>50	82 (36)
Postresidency position in July 2020	
Private practice	71 (31)
Facial plastics and reconstructive surgery fellowship	34 (15)
Head and neck fellowship	25 (11)
Rhinology fellowship	18 (8)
Pediatric fellowship	15 (7)
T32	13 (6)
Laryngology fellowship	12 (5)
Otology/neurotology fellowship	11 (5)
Academic general	10 (4)
Did not graduate	8 (4)
Academic‐affiliated	6 (3)
Sleep fellowship	2 (1)
Endocrine surgery fellowship	1 (<1)

Abbreviation: IQR, interquartile range.

^a^ Features were summarized with median (IQR; range) or n (%).

### Associations Between ERAS Application Features and Future Academic Potential

Associations between publication potential during residency and preresidency applicant features from ERAS are summarized in Table 4. Asian residents published significantly more than white residents (median 6 vs 4 publications, *p* = 0.002). Applicants who attended residency in the Northeast or West Coast and at the top 50 research output programs according to Doximity Residency Navigator published significantly more than residents at other programs (*p* < 0.001 for both). USMLE scores, AOA status, and the presence of advanced degrees were not significantly associated with increased publication potential during residency. The number of poster presentations, research experiences, and unpublished submissions before residency showed a statistically significant but weakly positive correlation with publication potential during residency.

**Table 4:** Associations WIth Number of Publications During Residency for 2014 to 2015 Applicants Who Matched and Graduated From ENT Residency, N = 205

Feature	Publications^a^	*p* value
Age at time of application (y)	−0.13	0.07
Sex		
Male	4 (2‐6)	0.32
Female	4 (2‐8)	
Race and ethnicity (N = 198)		
Asian	6 (3‐10)	**0.002**
White	4 (2‐6)	
All others	3 (1‐5)	
Couples match (N = 186)		
No	4 (2‐7)	0.48
Yes	3 (1‐6)	
Birthplace		
United States	4 (2‐6)	0.39
Outside of the United States	4 (2‐10)	
Region of US birthplace (N = 174)		
Northeast	5 (3‐7)	
Southeast	3 (2‐6)	
Southwest	2 (2‐4)	
Midwest	4 (2‐6)	
West	5 (2‐8)	0.11
US citizen		
No	6 (3‐10)	0.35
Yes	4 (2‐7)	
Region of US medical school		
Northeast	5 (3‐9)	<**0.001**
Southeast	4 (2‐6)	
Southwest	2 (1‐3)	
Midwest	3 (1‐6)	
West	6 (4‐9)	
USMLE Step 1	0.07	0.30
USMLE Step 2 (N = 174)	0.03	0.69
AOA		
No	3 (1‐6)	0.26
Yes	4 (2‐6)	
Not awarded at school	5 (2‐10)	
Advanced degree		
No	4 (2‐7)	0.68
Yes	3 (2‐8)	
Undergraduate field of study		
Sciences and health	4 (2‐7)	0.45
All others	3 (1‐6)	
Number of poster presentations	0.22	**0.002**
Number of oral presentations	0.13	0.07
Research experience	0.24	**<0.001**
Number of reported total publications	0.14	0.05
Number of reported first‐author publications	0.09	0.18
Number of reported unpublished submissions	0.27	**<0.001**
Region of residency		
Northeast	6 (3‐11)	<**0.001**
Southeast	3 (1‐6)	
Southwest	2 (1‐3)	
Midwest	4 (1‐6)	
West	5 (3‐8)	
Residency Doximity research ranking		
1‐20	6 (3‐10)	<**0.001**
21‐50	5 (3‐7)	
>50	2 (1‐4)	

Bolded values indicate statistical significance, defined as *p* < 0.05.

Abbreviations: AOA, Alpha Omega Alpha; IQR, interquartile range; USMLE, United States Medical Licensing Examination.

^a^ For continuous features, associations were summarized with Spearman rank correlation coefficients and associated *p* values. For categorical features, the number of publications during residency was summarized with medians (IQRs) and *p* values were obtained from Spearman rank correlation coefficients, Kruskal‐Wallis tests, or Wilcoxon rank sum tests.

Associations between future career paths and preresidency applicant features are shown in Table 5. The only features significantly associated with postresidency career plans were the age and sex of the applicant before residency. Graduates going straight into general academic or academic‐affiliated practice were younger (*p* = 0.026). The majority of female (n = 56, 74%) graduates entered fellowship, which was a significantly higher proportion compared to men (*p* = 0.002).

**Table 5:** Associations With Postresidency Positions in July 2020 for 2014 to 2015 Applicants Who Matched and Graduated From ENT Residency, N = 205

Feature^a^	Private (n = 71)	Academic (n = 16)	Fellowship (n = 118)	*p* value^b^
Age at time of application (y)	26 (25‐28)	25 (25‐26)	26 (25‐28)	**0.026**
Sex				
Male	55 (77)	12 (75)	62 (53)	**0.002**
Female	16 (23)	4 (25)	56 (47)	
Race and ethnicity (N = 198)				
Asian	11 (16)	3 (20)	28 (25)	0.57
White	49 (71)	9 (60)	73 (64)	
All others	9 (13)	3 (20)	13 (11)	
Couples match (N = *186*)	5 (7)	1 (9)	7 (6)	0.72
Birthplace				
United States	63 (89)	15 (94)	96 (81)	0.23
Outside of the United States	8 (11)	1 (6)	22 (19)	
Region of US birthplace (N = 174)				
Northeast	10 (16)	2 (13)	23 (24)	0.65
Southeast	11 (17)	2 (13)	16 (17)	
Southwest	5 (8)	0	11 (11)	
Midwest	23 (37)	6 (40)	28 (29)	
West	14 (22)	5 (33)	18 (19)	
US citizen	69 (97)	16 (100)	114 (97)	1.0
Region of US medical school				
Northeast	11 (15)	4 (25)	24 (20)	0.65
Southeast	18 (25)	4 (25)	40 (34)	
Southwest	9 (13)	0	9 (8)	
Midwest	25 (35)	6 (38)	33 (28)	
West	8 (11)	2 (13)	12 (10)	
USMLE Step 1	250 (243‐255)	250 (246‐256)	248 (242‐257)	0.69
USMLE Step 2 (N = 174)	252 (246‐260)	259 (253‐263)	259 (248‐265)	0.10
AOA				
No	36 (51)	5 (31)	48 (41)	0.52
Yes	28 (39)	9 (56)	53 (45)	
Not awarded at school	7 (10)	2 (13)	17 (14)	
Advanced degree	7 (10)	1 (6)	25 (21)	0.07
Undergraduate field of study				
Sciences and health	51 (72)	12 (75)	83 (70)	0.92
All others	20 (28)	4 (25)	35 (30)	
Number of poster presentations	2 (1‐4)	3 (0‐6)	2 (1‐4)	0.54
Number of oral presentations	1 (0‐2)	1 (0‐1)	1 (0‐3)	0.20
Research experience	4 (3‐6)	4 (3‐6)	5 (3‐6)	0.95
Number of reported total publications	0 (0‐2)	1 (1‐3)	1 (0‐2)	0.11
Number of reported first‐author publications	0 (0‐0)	0 (0‐1)	0 (0‐1)	0.72
Number of reported unpublished submissions	1 (0‐2)	1 (0‐2)	1 (0‐3)	0.80
Region of residency				
Northeast	9 (13)	7 (44)	29 (25)	0.34
Southeast	21 (30)	2 (13)	30 (25)	
Southwest	6 (8)	1 (6)	10 (8)	
Midwest	24 (34)	4 (25)	35 (30)	
West	11 (15)	2 (13)	14 (12)	
Residency Doximity research ranking				
1‐20	15 (21)	3 (19)	36 (31)	0.20
21‐50	26 (37)	5 (31)	42 (36)	
>50	30 (42)	8 (50)	40 (34)	

Bolded values indicate statistical significance, defined as *p* < 0.05.

Abbreviations: AOA, Alpha Omega Alpha; IQR, interquartile range; USMLE, United States Medical Licensing Examination.

^a^ Features were summarized with median (IQR) or n (%).

^b^ *p* values were obtained from Kruskal‐Wallis tests for continuous or ordinal features and *χ* ^2^ or Fisher exact tests for categorical features.

## Discussion

This is the first study examining the association between academic metrics provided on ERAS applications and the future publication potential and career trajectory of residents matching into otolaryngology. Overall, USMLE Step scores and AOA status were not predictive of resident publication records during training or enrollment in advanced fellowship after graduation. The number of research experiences and poster presentations before residency was associated with publication potential after the match. Asian residents and those who attended residency on the Northeast or West Coast had significantly more publications than other demographic groups. The only features that were significantly associated with enrollment in advanced fellowship training after residency graduation were the age and sex of the applicant before residency, with women significantly more likely to enter fellowship training than men. These findings indicate that traditional academic metrics used to screen applicants by many selection committees do not predict future publication potential.

Otolaryngology is a competitive specialty. In 2021, the overall match rate in otolaryngology was only 62%, with a match rate among US MD seniors of only 77%.^1^ In 2021, the average applicant applied to more than half of all US otolaryngology training programs.^3^ This is partially due to the decreased financial burden of the virtual interview interface adopted during the COVID‐19 pandemic. With the surge of applications per program, detailed application review is more time‐consuming. Often, it is easier for selection committees to fall back on traditional academic metrics to predict resident performance. A review in 2016 revealed that otolaryngology applicants had a higher USMLE Step 1 score (248 vs 233) and a higher proportion of AOA members (45% vs 17%) compared to all other specialties.^4^

However, the reliance on these academic metrics can in fact create biases in the selection process. While USMLE Step scores appear to predict performance on written boards,^14^ most academic otolaryngology faculty do not believe that it correlates with patient care or surgical skills.^5^ The poor discriminating ability of USMLE scores may lie in the high and narrow score range seen among matched applicants. While significantly low scores (ie, below 200) may predict a lower propensity for success in residency, the narrow range of scores among most applicants into otolaryngology makes this a nondiscriminating academic metric. In fact, the test was not designed or validated to be used to discriminate applicants performing at the highest end of the score distribution. Furthermore, when used for resident selection, USMLE scores can create sex and racial biases—women and underrepresented minorities on average have lower USMLE Step scores compared to their demographic counterparts.^15^ Similarly, only 2.2% of AOA members identify as black, and white students are much more likely to be selected for AOA membership than black students.^16^ As a result, an emphasis on these standard metrics could lead to further underrecruitment of women and underrepresented minorities into our field—one that struggles more than most surgical subspecialties in its recruitment of Black trainees.^17^ The search for other metrics to predict resident success is even more pressing in the setting of USMLE Step 1 going to a pass/fail system.^5^

Our results suggest that the volume of research experiences, not publications, is associated with research success during residency. These results may reflect the challenges that medical students face in seeing research projects to full publication. Longitudinal projects can take years to complete, and placing undue emphasis on the number of publications students should achieve may deter students from joining in on long‐term projects in favor of shorter retrospective studies or case reports. When it comes to research, it appears that the effort students put in, as measured by the number of experiences, is a better predictor of enduring success than the end product of publications.

Research experience too must be used conservatively in the selection process. In 2020, matched applicants into otolaryngology had an average of over 6 research experiences and over 13 abstracts, presentations, and publications.^18^ Yet research productivity early in training does not necessarily lead to a career as a clinician‐scientist. Clinical responsibilities during residency are time‐demanding, and there are many perceived obstacles to pursuing a career in research including a low salary, high competition for funding, and lack of departmental support.^19^ Outside of clinical responsibilities, residents may choose to engage in other academic pursuits such as quality improvement or leadership positions within their institution or national societies. With limited free time, residents may also choose to spend any time away from clinical responsibilities with family—caring for children or elders. Greater research productivity early in training may lead to greater burnout, which decreases research productivity later on. Moreover, a focus on research experience prior to residency can further discriminate against underrepresented minorities, as most academic centers do not offer funding to trainees seeking to take a research gap year and underrepresented minority students may already have less robust academic networks and financial resources.^17^ In fact, underrepresented minority students report significant difficulty in finding mentorship, particularly race‐concordant mentorship, within otolaryngology.^17^ Without mentorship, these students may face even greater challenges in gaining the preresidency research experience that some training programs may expect.

The association between demographic features and research productivity during otolaryngology residency appears to be novel to this study. Asian race was associated with a significantly higher number of publications during residency compared to Whites. Unlike other surgical subspecialties, Asians are overrepresented within the field of otolaryngology.^20^ As such, the drive for Asian residents to publish could be related to the “model minority” mentality^21^ and the perceived notion and pressure that career advancement in the field requires further academic success compared to their White counterparts. In reality, however, applicants who self‐identify as Asian via ERAS can come from culturally diverse and heterogeneous backgrounds—some of which may remain underrepresented. We also found that residents who trained or attended medical school in the Northeast or West had a significantly higher number of publications compared to those in other regions. This is likely related to the density of highly funded and productive research universities along the two coasts, as studies have shown that faculty at academically competitive universities are more productive than those in less competitive environments.^22^ In fact, in this cohort, residents at the top 50 research output training programs, per Doximity Residency Navigator, published significantly more than residents at other programs.

Finally, our results showed that women were significantly more likely than men to enroll in fellowship training, contradicting other studies on the representation of women in otolaryngology. This finding could simply be because more women than men are pursuing careers in academia. On the other hand, in 2017, although over 35% of trainees were women, women represented only 14.5% of practicing otolaryngologists.^23^ In 2020, there were only 5 female chairs in otolaryngology, representing only 5.1% of chair positions.^24^ The discrepancy between the poor representation of women in otolaryngology compared to the high proportion of women pursuing additional training could be attributed to the perception among women that additional training is necessary to gain respect from their male counterparts and to advance their careers. Nearly one‐fifth of women feel that their gender can hinder their career advancement.^23^ Given the low number of women in academic otolaryngology, fellowship training could be 1 avenue for women to pursue additional career mentorship. Finally, gender‐based discrepancies in surgical case volumes among otolaryngology residents may lead some women to feel that they did not gain sufficient experience during residency.^25^

The future of resident selection in otolaryngology must take on a more holistic approach. In addition to not predicting an applicant's clinical success, our results show that USMLE scores and AOA status also do not predict publication potential or future career trajectory. Furthermore, many of these academic requirements can discriminate against underrepresented minority students^17^ and widen the gap in gender and racial representation seen in this field. In recent years, many residency programs have changed their approach to the selection process, including “unblinded” application reviews to limit implicit bias and the adaptation of algorithms that do not solely rely on traditional metrics to identify strong diverse trainees^26^

This study is not without limitations. This is a retrospective study examining a single application cycle into otolaryngology, with no long‐term follow‐up and details regarding career trajectory after fellowship. Publication potential was measured only in the number of publications, and not in the impact of those publications, as the *H*‐index of most applicants could not be found. In addition, we recognize that publication potential only represents 1 facet of resident academic success during training. Success in research may not, and likely does not predict resident performance on board examinations, acquisition of surgical skills, clinical judgment, or bedside manner—all of which are equally important measures of competency in surgical training. We also do not advocate that future career trajectories in fellowship, academia, or private practice should influence a training program's decision to select applicants. In reality, the decision to enter a certain career trajectory is multifaceted, and mixed methods studies are likely needed to fully understand the motivations behind a trainee's career choice. Ultimately, programs should aim to select a diverse group of trainees with individualized career aspirations and provide excellent training to residents who should graduate well‐equipped to enter any practice environment that they desire. Finally, it is important to acknowledge that the retrospective associations between demographic factors and academic performance discovered in this study do not imply causality. They should not be used by programs as a reliable predictor of future success or used in selection algorithms going forward.

## Conclusion

In otolaryngology, not all preresidency academic metrics are associated with publication potential during residency or propensity for postresidency fellowship training. Research experience prior to residency is associated with greater research output during training, but USMLE scores, the total number of publications and AOA status are not. Programs should not use academic metrics alone to predict an applicant's future academic success or career trajectory. A diverse range of academic and life experiences should be considered in the selection of the next generation of otolaryngologists.

## Author Contributions

**Linda X. Yin**, conceptualization, methodology, literature review, data collection, data interpretation, writing, review and editing; **Dominic J. Catalano**, conceptualization, methodology, literature review, data collection, data interpretation, review and editing; **Susan E. Bisco**, methodology, data collection, data interpretation, review and editing; **Christine M. Lohse**, statistical analysis, writing, review and editing; **Matthew L. Carlson**, conceptualization, methodology, supervision, writing, review and editing; **Janalee K. Stokken**, conceptualization, methodology, supervision, writing, review and editing.

## Disclosures

### Competing interests

None.

### Funding source

Internal only.

## References

[1] National Resident Matching Program . Results and data—2021 main residency match (Report); 2021.

[2] Kaplan AB , Riedy KN , Grundfast KM . Increasing competitiveness for an otolaryngology residency: where we are and concerns about the future. Otolaryngol Head Neck Surg. 2015;153(5):699‐701. 26187905 10.1177/0194599815593734

[3] Pletcher SD , Chang CWD , Thorne MC , Malekzadeh S . The otolaryngology residency program preference signaling experience. Acad Med. 2022;97:664‐668. 34618735 10.1097/ACM.0000000000004441 PMC9028299

[4] Bowe SN , Schmalbach CE , Laury AM . The state of the otolaryngology match: a review of applicant trends, “Impossible” qualifications, and implications. Otolaryngol Head Neck Surg. 2017;156(6):985‐990. 28319452 10.1177/0194599817695804

[5] Goshtasbi K , Abouzari M , Tjoa T , Malekzadeh S , Bhandarkar ND . The effects of pass/fail USMLE Step 1 scoring on the otolaryngology residency application process. Laryngoscope. 2021;131(3):E738‐E743. 32880975 10.1002/lary.29072 PMC8051359

[6] Thompson RH , Lohse CM , Husmann DA , Leibovich BC , Gettman MT . Predictors of a successful urology resident using medical student application materials. Urology. 2017;108:22‐28. 28751165 10.1016/j.urology.2017.06.046

[7] Hauser LJ , Gebhard GM , Blumhagen R , Carlson NE , Cabrera‐Muffly C . Applicant characteristics associated with successful matching into otolaryngology. Laryngoscope. 2017;127(5):1052‐1057. 27767217 10.1002/lary.26236 PMC5392128

[8] Daly KA , Levine SC , Adams GL . Predictors for resident success in otolaryngology. J Am Coll Surg. 2006;202(4):649‐654. 16571437 10.1016/j.jamcollsurg.2005.12.006

[9] Vaughan LA , Quick JA . Evidence‐based selection of surgical residents. Surg Clin North Am. 2021;101(4):667‐677. 34242608 10.1016/j.suc.2021.05.012

[10] Chole RA , Ogden MA . Predictors of future success in otolaryngology residency applicants. Arch Otolaryngol Head Neck Surg. 2012;138(8):707‐712. 22911295 10.1001/archoto.2012.1374

[11] Mansi A , Karam WN , Chaaban MR . Attitudes of residents and program directors towards research in otolaryngology residency. Ann Otol Rhinol Laryngol. 2019;128(1):28‐35. 30371115 10.1177/0003489418804565

[12] Naclerio RM , Saengpanich S , Spainhour M , Baroody FM . The otolaryngology research paradox. Arch Otolaryngol Head Neck Surg. 2001;127(10):1181‐1184. 11587596 10.1001/archotol.127.10.1181

[13] Wright‐Chisem J , Cohn MR , Yang J , Osei D , Kogan M . Do medical students who participate in a research gap year produce more research during residency? J Am Acad Orthop Surg Glob Res Rev. 2021;5(5):e21.00061. 10.5435/JAAOSGlobal-D-21-00061 PMC8126556 33983156

[14] Velez DR . Prospective factors that predict American board of surgery in‐training examination performance: a systematic review. Am Surg. 2021;87:1867‐1878. 34763542 10.1177/00031348211058626

[15] Quesada PR , Solis RN , Ojeaga M , Yang NT , Taylor SL , Diaz RC . Overemphasis of USMLE and its potential impact on diversity in otolaryngology. OTO Open. 2021;5(3):2473974X2110314. 10.1177/2473974X211031470 PMC8295955 34350370

[16] Wijesekera TP , Kim M , Moore EZ , Sorenson O , Ross DA . All other things being equal: exploring racial and gender disparities in medical school honor society induction. Acad Med. 2019;94(4):562‐569. 30234509 10.1097/ACM.0000000000002463

[17] O'Brien EK , Douse DM , Bayan SL , Stokken JK , Van Abel KM . Increasing the number of black otolaryngologists. Otolaryngol Clin North Am. 2021;54(2):457‐470. 33743890 10.1016/j.otc.2020.11.017

[18] National Resident Matching Program . Charting outcomes in the match: senior students of U.S. MD medical schools (Report); 2020.

[19] Harris JP , Edstrom OE . Factors influencing a clinician‐scientist career path in otolaryngology. Laryngoscope. 2022;132:1555‐1560. 34773409 10.1002/lary.29940

[20] Kim Y , Kassam AF , McElroy IE , et al. The current status of the diversity pipeline in surgical training. Am J Surg. 2022;224:250‐256. 34776239 10.1016/j.amjsurg.2021.11.006

[21] Tu MC , Okazaki S . What is career success? A new Asian American psychology of working. Am Psychol. 2021;76(4):673‐688. 34410742 10.1037/amp0000807

[22] Way SF , Morgan AC , Larremore DB , Clauset A . Productivity, prominence, and the effects of academic environment. Proc Natl Acad Sci USA. 2019;116(22):10729‐10733. 31036658 10.1073/pnas.1817431116 PMC6561156

[23] O'Connell Ferster AP , Hu A . Women in otolaryngology. Otolaryngol Head Neck Surg. 2017;157(2):173‐174. 28485204 10.1177/0194599817706496

[24] Epperson M , Gouveia CJ , Tabangin ME , et al. Female representation in otolaryngology leadership roles. Laryngoscope. 2020;130(7):1664‐1669. 31532847 10.1002/lary.28308

[25] Gurgel RK , Cardon BR , Allen CM , et al. Evaluating gender parity in operative experience for otolaryngology residencies in the United States. Laryngoscope. 2020;130(7):1651‐1656. 31532842 10.1002/lary.28306

[26] Villwock JA , Hamill CS , Sale KA , Sykes KJ . Beyond the USMLE: the STAR algorithm for initial residency applicant screening and interview selection. J Surg Res. 2019;235:447‐452. 30691828 10.1016/j.jss.2018.07.057

